# Supplementary material for: Boosting functionality of synthetic DNA circuits with tailored deactivation
Source: Nat Commun. 2016 Nov 15;7:13474. doi: 10.1038/ncomms13474 (PMC5116077; doi:10.1038/ncomms13474)
Supplement: Supplementary Information — Supplementary Figures 1-10, Supplementary Table 1, Supplementary Notes 1-3, Supplementary Methods, Supplementary References. [file ncomms13474-s1.pdf]

Supplementary Information

Supplementary Figures

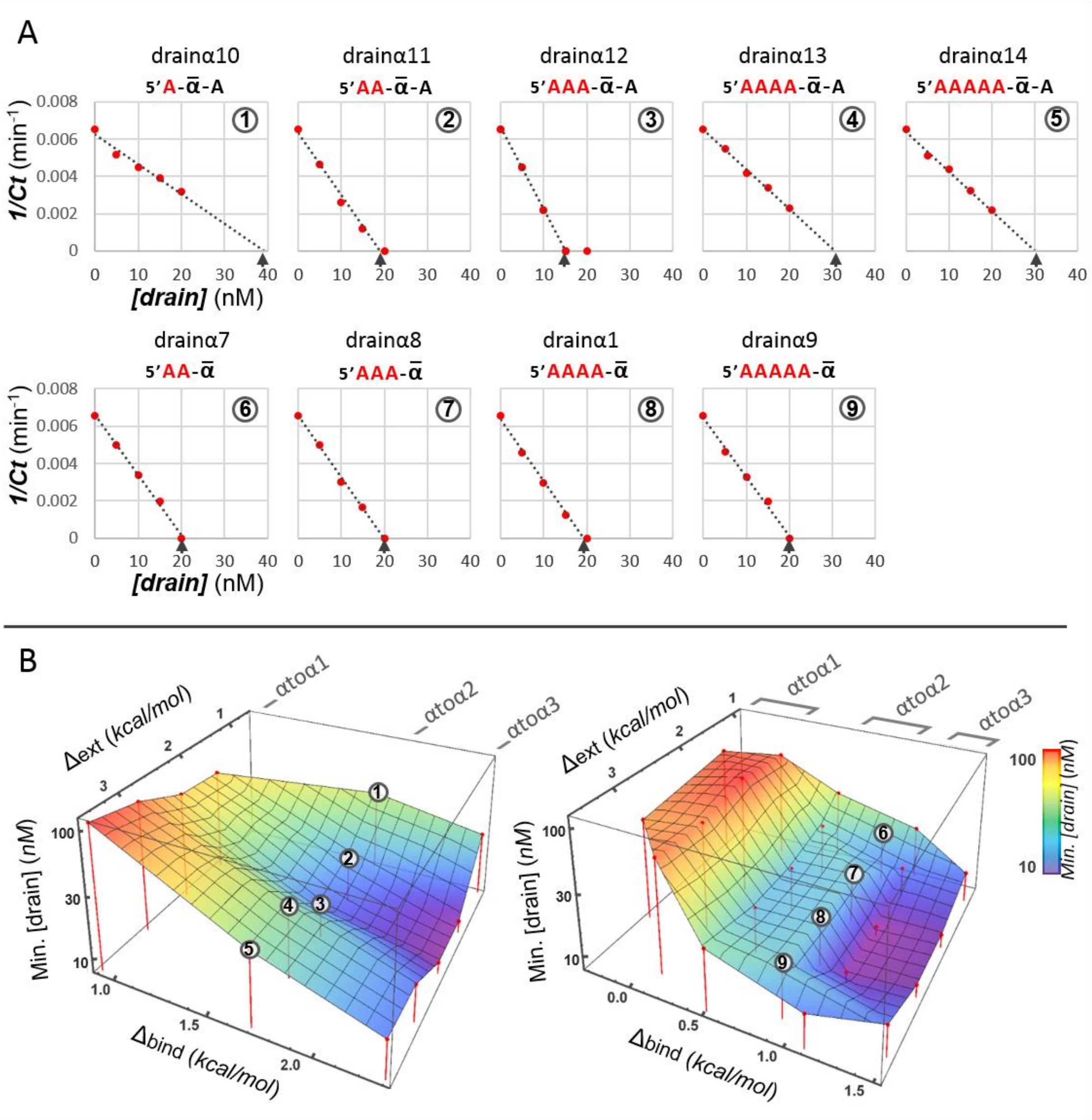

Supplementary Figure 1: ***Drain template efficiency as a function of thermodynamic parameters (Δ_{bind}, Δ_{ext}).*** A) Experimental determination of the minimal drain concentration required to reach the bistable regime (Min. [drain]). 50 nM of the autocatalytic template *α*to*α*2 are incubated with varying concentrations of drain and the time for the amplification to reach 20% of its maximum amplitude (Ct) is measured. The linear regression of the plot 1/*Ct*= f([drain]) allows

the direct estimation of Min. [drain] for each drain template (arrows). The experiment is reproduced for the templates *α*to*α*1 and *α*to*α*3 with various drain templates (data not shown, cf. Supplementary Table 1B for sequences). B) Min. [drain] is plotted in the $\Delta_{ext}/\Delta_{bind}$ parameter space for the drains with (left) or without (right) an additional 3' dA.

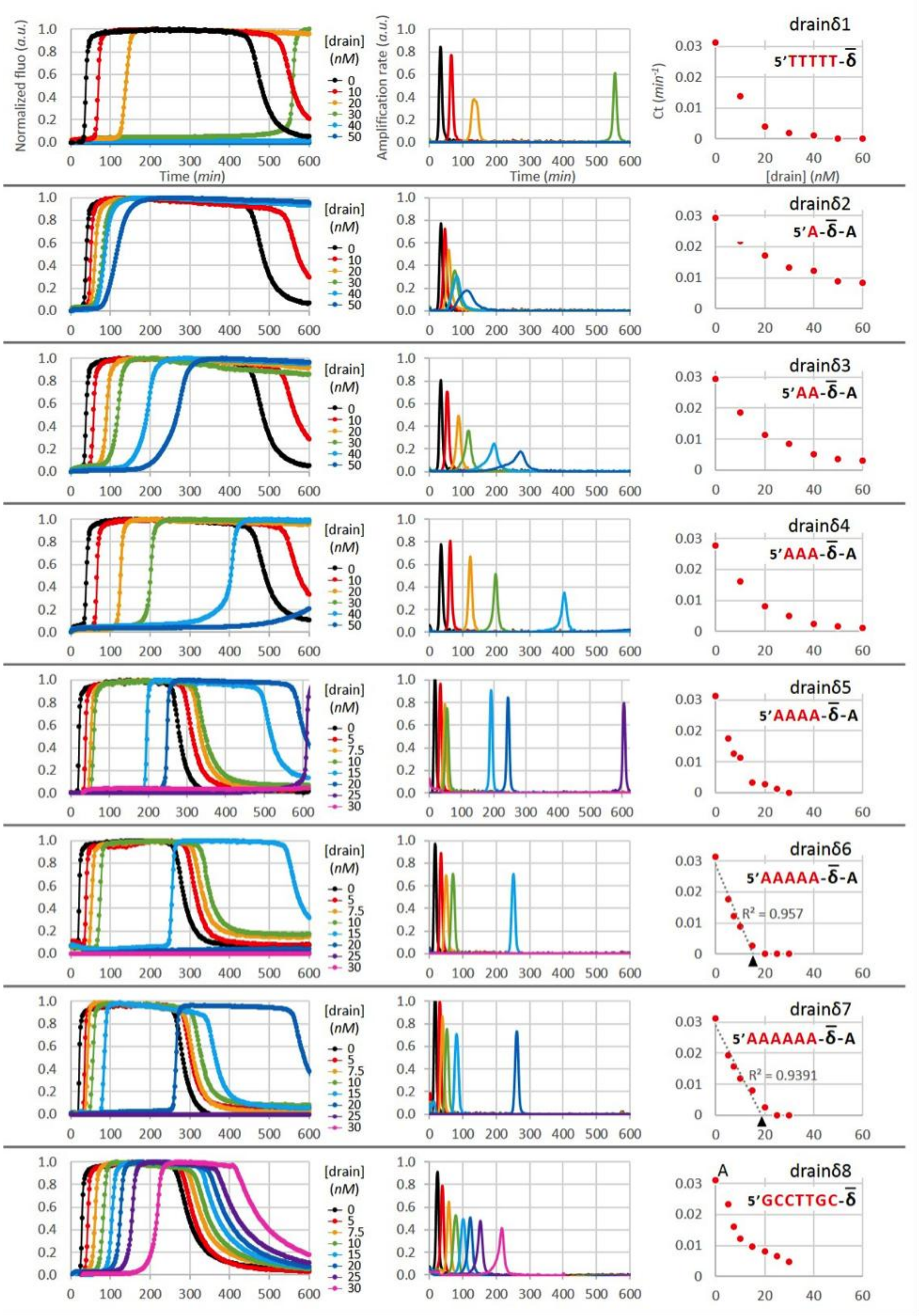

Supplementary Figure 2: ***Implementation of the drain-based bistability mechanism with the nicking enzyme Nt.BstNBI.*** The amplification of the autocatalyst *δ*to*δ* (50 nM) is monitored with the double-strand-specific fluorophore EvaGreen (left column). The replication template is

incubated with various concentrations of various drain templates (drainδ1-8) with different extensions. All sequences and thermodynamic parameters are reported in Supplementary Table 1. The amplification rate for each experimental time trace (middle column) is calculated as the derivative of the normalized recorded fluorescence. 1/*Ct* is plotted as a function of the drain template concentration (right column). When reasonable, a linear regression allows the direct estimation of Min. [drain] (arrows).

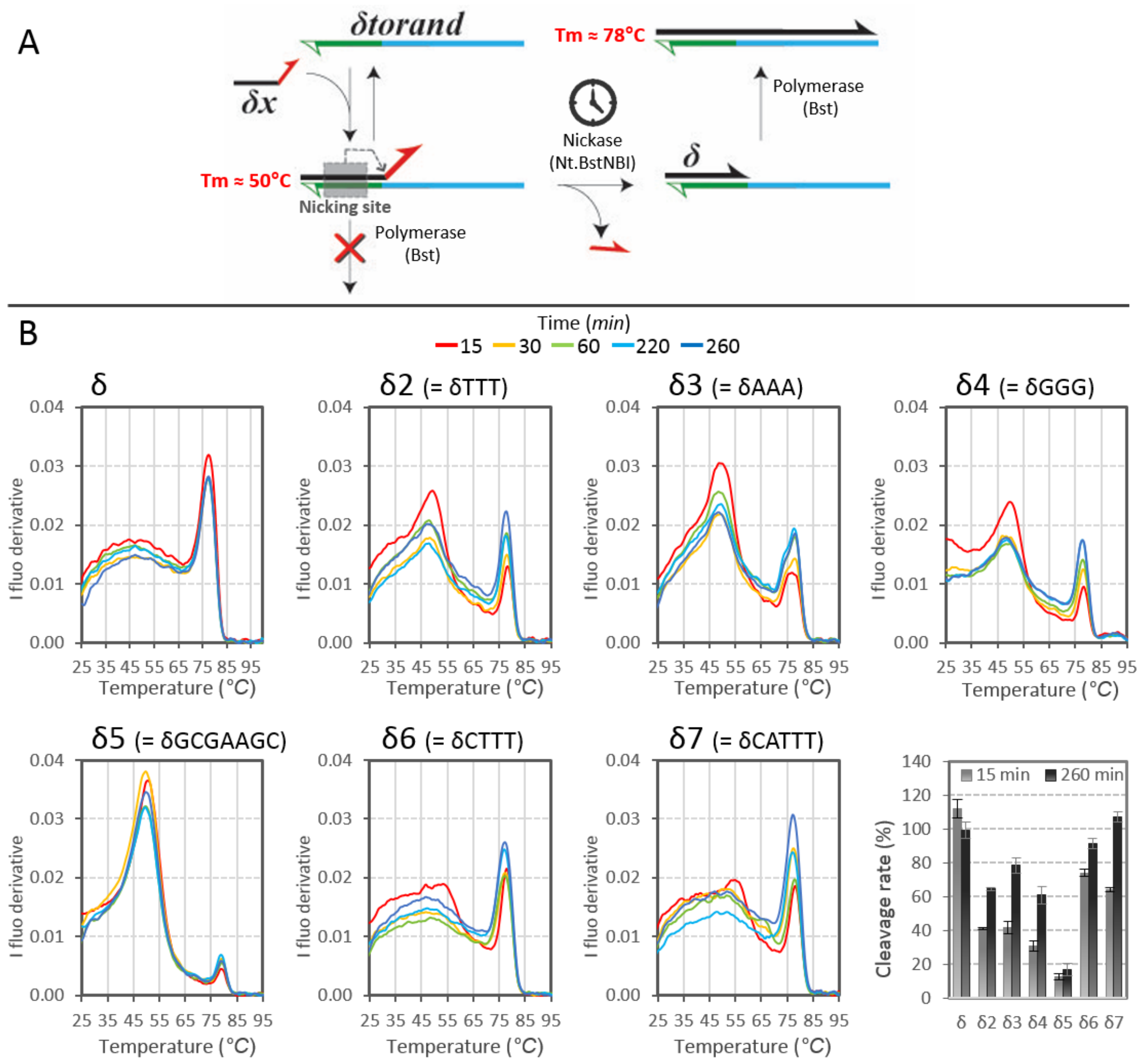

Supplementary Figure 3: ***Reactivation of extended triggers by Nt.BstNBI.*** In order to test the leak mechanism discussed above, we designed an indirect assay to assess the nicking efficiency of Nt.BstNBI working on triggers extended with mismatches (A). Template *δ*torand, whose 3’ part is complementary to the δ sequence, is used as a nicking site-containing template, with a primer binding site complementary to the trigger sequence. First, the template is incubated with an equimolar amount of *δ* or triggers extended by various tail sequences (*δ*2-*δ*7). At the beginning of the run, Nt.BstNBI is added and the reaction is quenched at different times by heat inactivation. Then, the polymerase *Bst full length* is added to extend matched triggers (which would result from the nicking of mismatched triggers). Finally, a melting experiment reveals the relative proportion of unnicked triggers (Tm ≈ 50°C) and nicked/polymerized triggers (Tm ≈ 78°C). (B) This method enables the semi-quantitative determination of the cleavage rate occurring during the first step (the protocol is detailed in the experimental section). As a positive control,

the addition of the correct, matched trigger *δ* to the reaction mixture leads to its quantitative elongation along *δ*torand (a single product melting at 78 °C). Similarly, the trigger extended with one (*δ*6) or two (*δ*7) matching bases followed by three mismatches is efficiently cut by the nickase and quantitatively elongated for incubation times over 30 minutes. Triggers exhibiting a mismatched extension are nicked less efficiently but this reaction still occurs, as demonstrated by the spike intensity of the extended products increasing over time. The cleavage for *δ*2-4 is 30-40 % after 15 minutes and approximates 60-80 % after 260 minutes. Therefore the rate on mismatched substrates is approximately half the rate on matched substrates. The tail composition (TTT, AAA or GGG) does not seem to affect nicking efficiency. Only *δ*5, extended by the ultraloop sequence GCGAAGC seams a poor substrate for Nt.BstNBI, suggesting steric protection of the nicking site by the double-stranded structure of the tail. Unfortunately, this secondary structure is also likely responsible for the reduced Δ_{ext} of the cognate drain templates, making the latter ineffective.

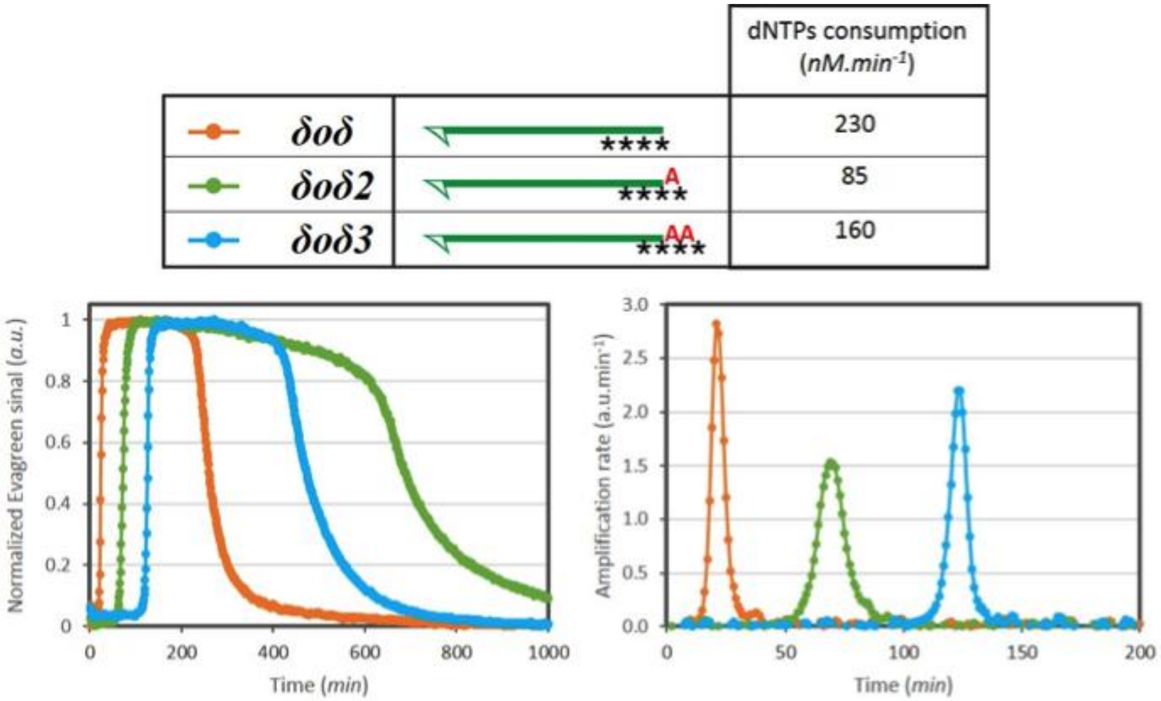

Supplementary Figure 4: *The nicking enzyme Nt.BstNBI runs a positive feedback loop even with 3' mismatched substrates.* To confirm the Nt.BstNBI-mediated reactivation mechanism hypothesis, we designed two replication templates modified with an extension of 1 (*δ*to*δ*2) or 2 (*δ*to*δ*3) dA at their 5' end (i.e. output side). This should prevent autocatalysis, because the output of the reaction is now a tailed form of the input that cannot be used for further priming (note the nicking site on the output side of the template contains a dU, so it is not active). These templates are incubated with the 3 enzymes of the PEN DNA toolbox supplemented with dNTPs and the amplification reaction is monitored in real-time with EvaGreen. The positive control, using an unmodified dual-repeat (*δ*to*δ*) self-starts and amplifies exponentially as expected. The signal then goes back to the baseline when all dNTP have been consumed and converted to dNMP. For *δ*to*δ*2-3, a delay is observed at the onset of the reaction; however, these lengthened templates eventually display a similar time trace, despite some slowing down of the dNTP consumption rate. This strongly supports the reactivation pathway resulting from the nicking of extended primers. The overall slowing down could then be attributed to the fact that two nicking events are now necessary in each autocatalytic cycle (one to reactivate the extended primer, one to liberate the product). We noticed that the positive feedback loop is less affected with the template exhibiting 2 additional nucleotides compared to the one modified with a single base extension. This observation suggests the nickase has a higher catalytic efficacy and/or affinity for triggers displaying longer mismatched extensions, or that the polymerase extends a fraction of the single mismatch triggers.

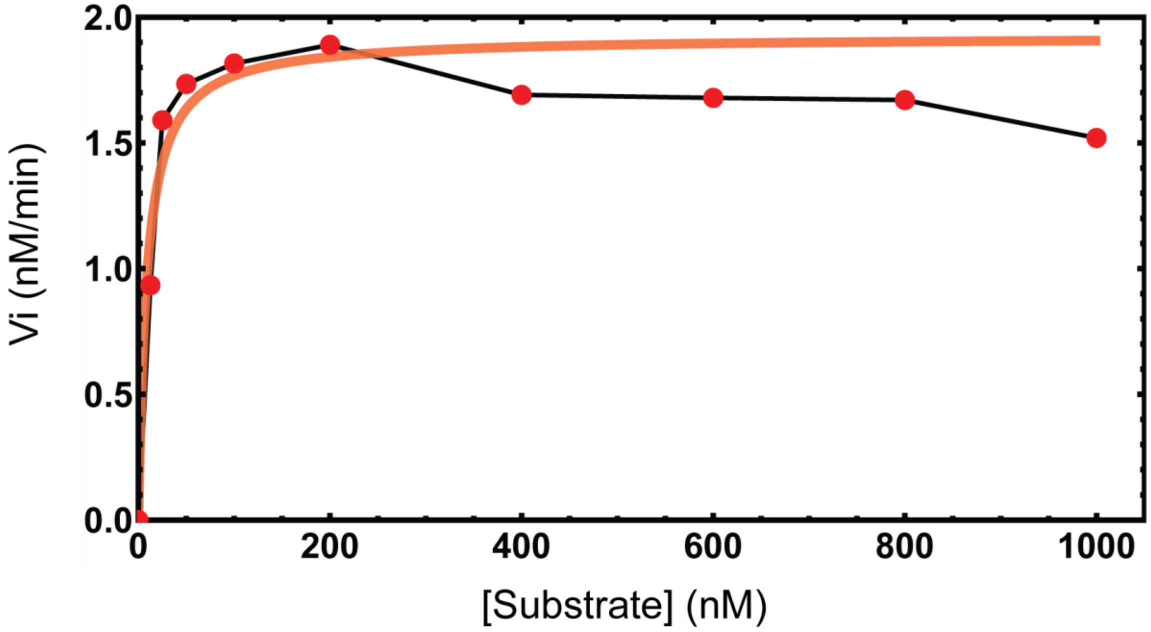

Supplementary Figure 5: ***Michaelis-Menten plot for the nicking enzyme Nb.BsmI at 10 U mL^{-1}.*** The enzyme was incubated a 45 °C in buffer with various quantities of a fluorogenic hairpin substrate (Cy5-*G*T*C*A**GAATG-C**TCG–AGAC-TTTTT-GTCT-CGA**G-^CATTC**TGAC-BHQ2; the recognition site in bold and the nicking site is indicated by ^; * are phosphorothioate modifications), and the initial rate was monitored by comparison with a standard. For fitting to the Michaelis-Menten equation (orange curve), the last 3 points were omitted, because they seemed to be affected by some form of non-Michaelian inhibition. The fitted parameters were $Vm_{@10U.mL^{-1}} = 1.9 nM.\min^{-1}$ and *Km* = 9 nM

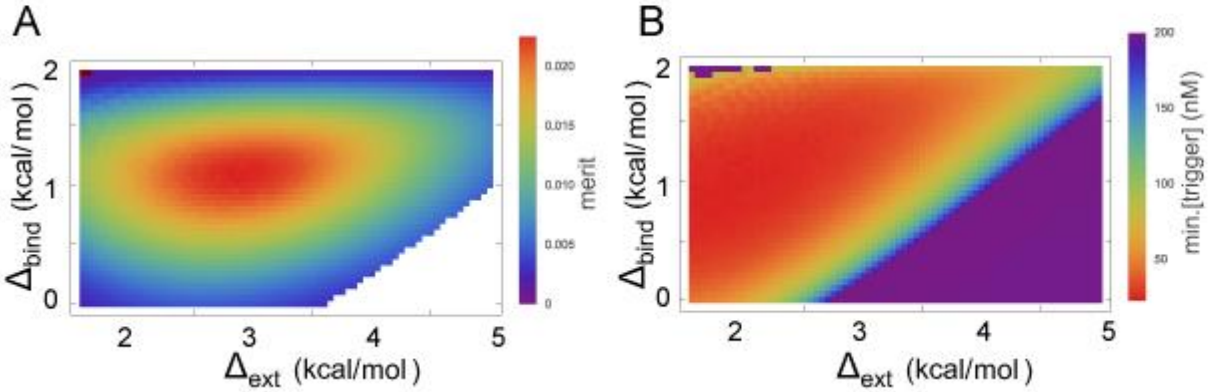

Supplementary Figure 6: ***Theoretical characterization of the drain-based bistable system.*** A) Figure of merit for a bistable system design, where one desires a responsive system that is reasonably loaded in templates (concentration cut-off is set at 50% of the concentration of autocatalytic templates), B) Minimum concentration of trigger required to switch from a low stable state to the high state, for various designs, assuming that the trigger is provided to the system as a one-step injection at time 0 (where all other dynamic concentrations are set to 0).

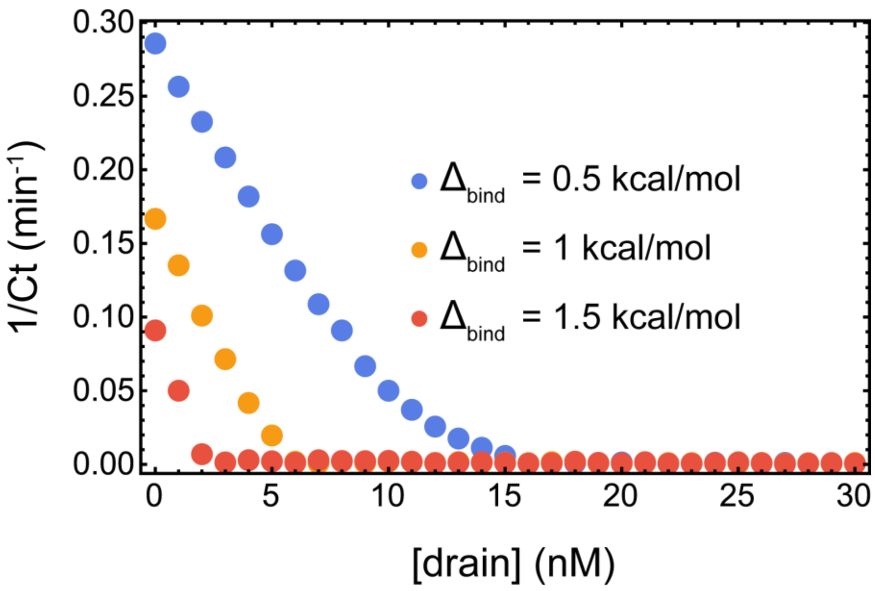

Supplementary Figure 7: ***Simulated plots of 1/Ct (where Ct is the time at which the autocatalytic species crosses a defined concentration) as a function of drain concentration, for various designs of the template/drain system.*** The plots shown are computed with Δ_{ext} = 2.0 kcal mol^{-1}. The linear relationship between *1/Ct* and the drain concentration reflects the sudden bifurcation to the bistable state. Note that simulations with a smaller Δ_{bind} show a less sharp transition, possibly because the drain concentration does not anymore directly control the draining rate.

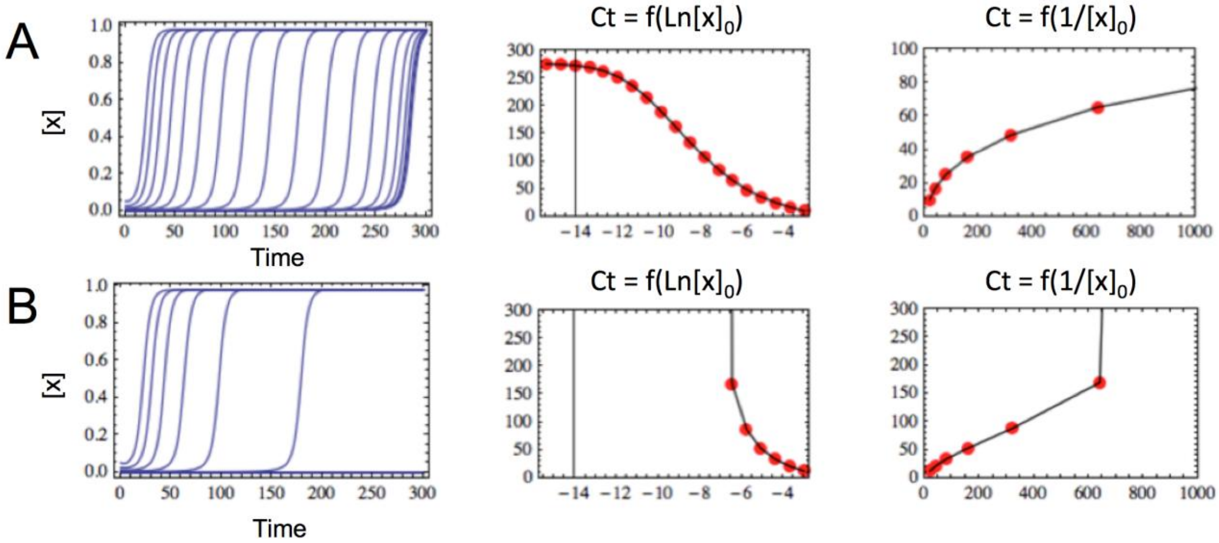

Supplementary Figure 8: ***Amplification curves and Ct values for various initial conditions $x_{t=0}$*****.** A) below and B) above the bistable threshold.

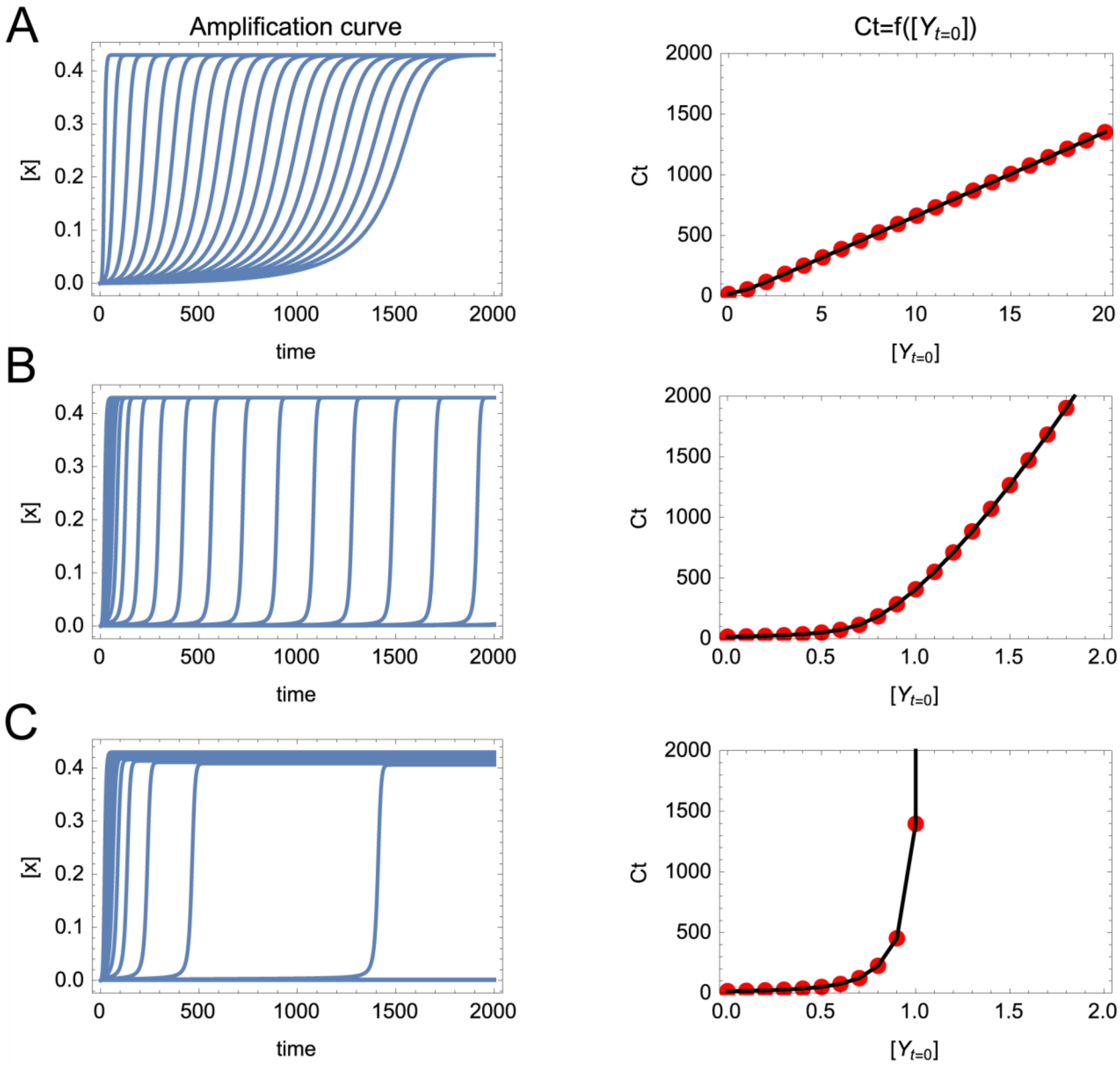

Supplementary Figure 9: ***The three scenarios for stabilizing the null state of an autocatalytic loop.*** A) Amplification curves, as well as Ct values, for various concentrations of a competitive binder (scenario 1). Model values are $k_{\text{leak}} = 3.10^{-3}$, $a = 1$, $b = 0.7$, $c = 0.5$, $c' = 0.1$. B) Amplification curves, as well as *Ct* values, for various concentrations of a suicide inhibitor (scenario 2). Model values are $k_{\text{leak}} = 3.10^{-4}$, $a = 1$, $b = 0.7$, $c = 0.5$. C) Amplification curves, as well as Ct values, for various concentrations of an irreversible and catalytic drain template (scenario 3). Model values are $k_{\text{leak}} = 3.10^{-4}$, $a = 1$, $b = 0.7$, $c = 0.5$, $c' = 5.10^{-3}$. The drain approach leads to bistability above a critical concentration.

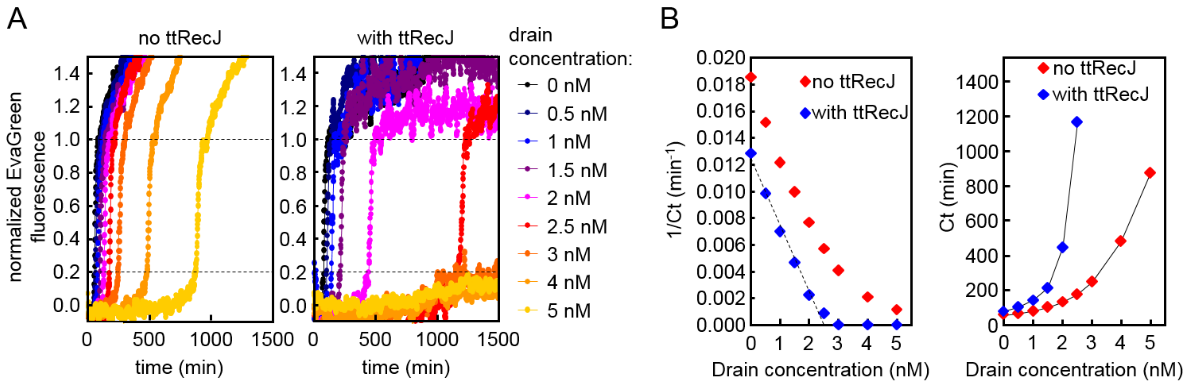

Supplementary Figure 10: *Experimental observation of the irreversible catalytic and non-catalytic scenarios.* A) The amplification of the autocatalyst *γtoγ* (50 nM) in the absence (left) or presence of ttRecJ (62 nM) and the indicated amounts of drainγ2 is monitored with EvaGreen. B) *1/Ct* values (left) and *Ct* values (right) plotted against drain concentration; the linear relationship between *1/Ct* and the drain concentration seen in the presence of ttRecJ is lost without the exonuclease; the curves on the right closely resemble those in Supplementary Fig. 9 regarding the irreversible non-catalytic (no ttRecJ) and catalytic (with ttRecJ) scenarios.

Supplementary Tables

A

NAME	SEQUENCE (5' -> 3')	Δ_{bind}	Δ_{ext}
αtoα1	C*T*C*GTCA**GAATGC**TCGTCAGAATG-P	-	-
αtoα2	C*T*C*G*TCA**GAATGC**TCGTCAGAAT-P	-	-
αtoα3	C*T*C*G*TCA**GAATGC**TCGTCAGAA-P	-	-
drainα1	A*A*A*A*CTCGTCAGAATG-P	0.2/1.0/1.6 †	2.6
drainα2	T*C*T*CGTCAGAATG-P	-0.2/0.6/1.2 †	0.9
drainα3	T*T*C*TCGTCAGAATG-P	-0.2/0.6/1.2 †	1.3
drainα4	T*T*T*CTCGTCAGAATG-P	-0.2/0.6/1.2 †	2.0
drainα5	T*T*T*TCTCGTCAGAATG-P	-0.2/0.6/1.2 †	2.7
drainα6	T*T*T*T*TCTCGTCAGAATG-P	-0.2/0.6/1.2 †	3.5
drainα7	A*A*C*T*CGTCAGAATG-P	0.2/1.0/1.6 †	1.1
drainα8	A*A*A*C*TCGTCAGAATG-P	0.2/1.0/1.6 †	1.9
drainα9	A*A*A*A*ACTCGTCAGAATG-P	0.2/1.0/1.6 †	3.3
drainα10	A*C*T*C*GTCAGAATGA-P	1.0/1.8/2.4 †	0.4
drainα11	A*A*C*T*CGTCAGAATGA-P	1.0/1.8/2.4 †	1.2
drainα12	A*A*A*C*TCGTCAGAATGA-P	1.0/1.8/2.4 †	1.9
drainα13	A*A*A*A*CTCGTCAGAATGA-P	1.0/1.8/2.4 †	2.6
drainα14	A*A*A*A*ACTCGTCAGAATGA-P	1.0/1.8/2.4 †	3.3
γtoγ2	C*G*A*TCCT**GAATGC**GATCCTGAA-P	-	-
drainγ2	A*A*A*-CGATCCTGAATG-P	1.6 ‡	1.9

† from left to right, Δ_{bind} are calculated for the templates αtoα1, αtoα2 and αtoα3

‡ Δ_{bind} is calculated for the template γtoγ2

B

NAME	SEQUENCE (5' -> 3')	ΔrG_1	ΔrG_2	ΔrG_3
δtoδ	Bioteg*C*A*A*TGACUCCTGCAAT**GACTC**C-P	-8.2	-	-
δtoδ2	Bioteg*A*C*A*ATGACUCCTGCAAT**GACTC**C-P	-8.2	-	-
δtoδ3	Bioteg*A*A*C*AATGACUCCTGCAAT**GACTC**C-P	-8.2	-	-
drainδ1	T*T*T*T*TCAATGACUCCTG-P	-	-11.8	-15.9
drainδ2	A*C*A*ATGACUCCTGA-P	-	-13.1	-13.4
drainδ3	A*A*C*AATGACUCCTGA-P	-	-13.1	-14.3
drainδ4	A*A*A*CAATGACUCCTGA-P	-	-13.1	-15.2

drainδ5	A*A*A*ACAATGACUCCTGA-P	-	-13.1	-16.0
drainδ6	A*A*A*AACAATGACUCCTGA-P	-	-13.1	-16.9
drainδ7	A*A*A*AAACAATGACUCCTGA-P	-	-13.1	-17.8
drainδ8	G*C*T*TCGCCAATGACUCCTG-P	-	-11.8	-22.7
δtorand	CATCTGGAAACCGTAATCCAACTTATTTTGCAATGACTCCTG	-9.8	-	-
δ	CAGGAGTCATTG	-	-	-
δ2	CAGGAGTCATTGTTT	-	-	-
δ3	CAGGAGTCATTGAAA	-	-	-
δ4	CAGGAGTCATTGGGG	-	-	-
δ5	CAGGAGTCATTGGCGAAGC	-	-	-
δ6	CAGGAGTCATTGCTTT	-	-	-
δ7	CAGGAGTCATTGCATTT	-	-	-

Supplementary Table 1: ***Sequences and thermodynamic values*** A) for the Nb.BsmI system and B) for the Nt.BstNBI system. Δ_{bind}, Δ_{ext}, ΔrG_1, ΔrG_2 and ΔrG_3 are all expressed in kcal mol^{-1}. * indicates a phosphorothioate bond. The Nb.BsmI and Nt.BstNBI recognition sites are in bold. P indicates a 3’ phosphorylation, and bioteg, a biotin-TEG modification.

Supplementary Notes

Supplementary Note 1: Network design

1.1 General design

The considerations given in Fig. 1 and 2 of the main text translate into a number of general design rules for a drain template aimed at stabilizing the 0 state of a positive feedback loop (and hence lead to bistability):

- At low concentrations, the reaction with the drain template must be the favored pathway. This is promoted by a binding energy in favor of the drain template.
- The inactivated (elongated) input must be released from the drain template at some rate. This is because the enzymatic sink used in this system, ttRecJ, is a single strand specific exonuclease, thus acting on dynamic DNA species only (or very predominantly) when they are free in solution. Failing to fulfill this condition would lead to a progressive poisoning of the pathway, ultimately rendering it useless once all the drain templates are in stable double-stranded form.
- It is important that templates with a missing base on their input site cannot be re-extended to their full length when bound to a trigger (and using it as an elongation template). This is ensured by having a phosphate 3' modification at this position (the empty arrows in Fig. 1 of the main text).
- There should be no pathway leading to the reactivation of the deactivated trigger, neither when it is still bound to the drain template, nor when it has been released in solution. This leads to different constraints depending on the nicking enzyme used, which are discussed in the next sections.
- Finally, note that since the basin of attraction of the null state is typically small (see Fig. 2 in the main text), departure from this state is a phenomenon that involves low trigger concentrations. Hence it is controlled by the linear regime of the enzymes, and we do not expect polymerase competition (between the template and the drain template) to play a significant role in the transition to bistability.

1.2 Design with the nicking enzyme Nb.BsmI (G^CATTC)

We have seen in the main text that a high Δ_{bind} is desirable, but that obtaining this at the expense of the template's input site stability leads to decreased amplification rates. Another option is instead to increase the stability of the drain template itself. One such strategy takes advantage of

the relatively strong dangle energies for some base configurations. Adenine is the base that tends to increase the most the stability of a DNA duplex through 3' dangle stacking, with energies equal to -0.42, -0.92, -0.82 and 0.12 kcal mol^{-1} for respectively 3'AT, 3'AG, 3'AC and 3'AA in standard conditions (1 M NaCl, pH = 7 and 37 °C)[1,2]. Other options would include the usage of modified bases with increased binding energies (2-amino-deoxyadenosine, aminoethyl-phenoxazine-deoxycytidine, 5-methyl-deoxycytidine, C-5 propynyl-deoxycytidine, C-5 propynyl-deoxyuridine, ...) in the drain template, but this was not tested.

We therefore designed two series of drains for the system *α*, with and without an additional 3' flanking deoxyadenosine and with tailing extensions of variable lengths (Supplementary Fig. 1). All these drains were tested with the amplification template short of 0, 1 or 2 bases. We then measured the time for the amplification to reach 20% of its maximum amplitude (Ct). The critical drain concentration was obtained by extrapolating the 1/Ct vs drain concentration curve (noting that in some cases, for *α*to*α*1 in particular, this would be associated with a large uncertainty on the critical value). This allowed us to reconstitute an experimental plot of drain effect in the $\Delta_{bind}/\Delta_{ext}$ plane. Comparison with the theoretical predictions (Fig. 2E and 2F in the main text; note that only the case with no flanking dA can be quantitatively compared because we define Δ_{bind} as a *destabilization* from the basic sequence) provides the following insights:

- The expected trends are globally reproduced by the experimental data, in particular for drains with 3'dA, where drain efficiency at stabilizing the 0 state clearly increases with increasing Δ_{bind} and decreasing Δ_{ext}. In the absence of 3'dA, Δ_{ext} seems to have less influence. As a consequence, a large variety of drain designs can be used to obtain bistability.
- As anticipated, drains with a very short extension (i.e. less than three bases) perform worse than thermodynamically predicted, possibly for one or more of the three following reasons: i) triggers with short mismatched extensions can still be extended by the polymerase on their amplification template; ii) drain/trigger duplexes with very short single strand extension are poor substrates for the polymerase; iii) the presence of phosphorothioates adjacent or within the binding site decreases the binding affinity (it is known that phosphorothioates depress the melting temperature of oligonucleotide duplexes by 1~2ºC per modification)[3].
- For long enough extensions, the hybridization thermodynamics generally controls the behavior but is not perfectly reflected (for example, ACC is more efficient than AGA in Fig. 3B; also poly-T 5'tail series with various Δ_{ext} perform better than poly-A tail series, even if they have lower Δ_{bind}), suggesting some possible sequence-specific effects.
- Additional stabilization of the drain template (here with a 3'dA) can decrease the amount of drain template necessary for stabilization of the low state, but tail length has to be adapted.

1.3 Systems based on the nicking enzyme Nt.BstNBI (GAGTCNNNN^)

To assess the versatility of drain templates to tune non-linearities of DNA-programmed nodes, we also tested a PEN amplification process relying on the nicking enzyme Nt.BstNBI. The relative arrangement of the recognition and cleavage site is different between Nt.bstNBI and Nb.BsmI, the nickase used in the main paper. For Nt.bstNBI, the recognition site of the nickase is within the sequence of the input and the nicking position is 4 nucleotides downstream of the recognition site. As a consequence, the site has to be deactivated on the drain sequence (if not, then the extended product on the drain template could be nicked back to its original trigger length). It has been shown that this is obtained by a dT to dU mutation on the bottom strand of the recognition sequence[4], therefore all drains were designed with this mutated sequence.
Also one should be careful that the nicking enzyme Nt.BstNBI cannot efficiently process blunt recognition sites. In our hands, at least one closed GC or CG pair is required 5' of the GAGTC recognition site (in the case of an open terminal AT pair we found that the dangle energy brought by an additional mismatched or dangling A led to correct nicking rates). This constraint limits the number of bases that can be removed from the input side of the template without affecting the nicking rate.
Supplementary Fig. 2 displays an array of experiments where a replication template δtoδ1 is incubated with different concentrations of drain template with various designs (drainδ1-8, cf. Supplementary Table 1B). The amplification is monitored in real-time with the double-strand specific intercalating dye EvaGreen. No trigger is introduced in the initial reaction mix. As with the system running on Nb.BsmI, the 0 state of the simple autocatalytic loop with degradation is unstable without drain template. However, our first try candidate, drainδ1 (which extends the trigger with a penta-adenylate tail), was able to stabilize the 0 state when present in relatively large amounts (>40 nM for 50 nM of δtoδ). By comparison, in the case of Nb.BsmI, for the same concentration of replication template αtoα, the bistable state is reached with less than 10 nM of drainα2 in the optimized experimental conditions.
Two reasons may explain the lower efficiency of the sink-based strategy with the Nt.BstNBI nicking enzyme. First, the Nt.BstNBI enzyme catalyzes the nicking step faster than Nb.BsmI in fast turnover conditions. Indeed, in experiments running with a limited amount of dNTP (e.g. 50 µM each), dNTP exhaustion is usually reached before 500 minutes with Nt.BstNBI, while it requires more than 1000 minutes with Nb.BsmI. Therefore, it is also possible that the leak rate, responsible for the self-start of the autocatalytic loop, is higher with Nt.BstNBI. Second, because of the relative arrangement of recognition and cleavage sites, the elongated input – resulting from polymerization along a drain template – still contains the recognition site in one case (Nt.BstNBI) and not in the other. Consequently, extended triggers, after their release from the

drain, could be reactivated by binding to the production template and having their tail nicked away by the endonuclease. Indeed, we demonstrated that the nickase Nt.BstNBI is able to cleave an oligonucleotide containing its recognition site, even if the sequence downstream of the nicking site is completely mismatched and single-stranded. This process appears to have little dependence on the mismatched bases that compose the tail, and the rate on a mismatched substrate is approximately 50 % of the rate on a matched one (Supplementary Fig. 3). Supplementary Fig. 4 further supports the existence and efficiency of this mechanism and shows that an autocatalytic replication template modified by a 5' extension – hence generating products that have additional mismatched bases when they migrate to the input site of the template – is still able to support an efficient autocatalytic loop. This reactivation mechanism of deactivated triggers by Nt.BstNBI is likely responsible for the weaker effect of drain*δ*1 compared to drains used with Nb.BsmI (the nicking site of Nb.BsmI is within the recognition site, hence the trigger does not carry an intact site).

We further attempted to increase the efficiency of the drain by optimizing its sequence. We also designed a set of oligonucleotides modified with a 3'-adenylate residue as above (hence increasing the Δ_{bind} from 2.6 kcal mol^{-1} for drain*δ*1 to 3.3 kcal mol^{-1} for drain*δ*2-7). From drain*δ*2 to drain*δ*7, the 5'-tail is composed of 1 to 6 dA, respectively, which define a broad range of Δ_{ext}. Their relative efficiency is evaluated in Supplementary Fig. 2. For the drains drain*δ*2-4, we noticed a sharp decrease of the amplification rate as a function of drain template concentration. This result is in agreement with the ODE model: qualitatively, a drain template with a low Δ_{ext} is able to deactivate triggers expeditiously because it is not limited by the dehybridization step. However, drains drainδ2-4 (Δ_{ext} < 3 kcal mol^{-1}, Δ_{bind} = 2.8 kcal mol^{-1}) performed poorly in delaying the self-start. We can hypothesize that the Nt.BstNBI-mediated reactivation pathway is responsible for this inefficiency, due to the high release rate of elongated triggers by the drain template. It is also possible that the polymerase is still able to extend triggers that carry only a short mismatched extension, as mentioned in section 1.2. For longer tails, the bistable regime is reached when enough drain templates are included in the mix. Drain*δ*5, drain*δ*6 and drain*δ*7 stabilize the 0 state below 30, 20 and 25 nM, respectively. For drain*δ*6 and drain*δ*7, the linear section of the plot 1/*Ct* = f([drain]) allows the accurate determination of the bistability threshold, which is 15 nM for drain*δ*6 and 19 nM for drain*δ*7. This result demonstrates the existence of an optimal drain template design with well-balanced $\Delta_{ext}/\Delta_{bind}$. This optimum may however depend on the working conditions, such as the temperature. As expected, these drain templates with relatively long tails do not competitively slow down the maximum amplification rate, since elongated triggers are very slowly released from drain templates (Δ_{ext} > 3 kcal mol^{-1}, Tm > 52°C).

In an attempt to limit the trigger reactivation leak, we also tested tails with some secondary structure. It has previously been shown that the short hepta-deoxyribonucleotide d(GCGAAGC) exhibits a stable stem-loop structure[5]. Indeed, we demonstrated that this particular extension significantly reduces the nicking rate of the trigger with a mismatched extension, $\delta 5$ (Supplementary Fig. 3). We thus designed the corresponding drain template (drain$\delta 8$), which drives the addition of that tail to the 3' end of δ. We hoped that the reduced reactivation rate would translate into an increased efficiency of drainδ8. However, although the nonlinearity of the amplification process is tuned as a function of drain$\delta 8$ concentration, the bistable regime was not observed (Supplementary Fig. 2, bottom). It is noteworthy that the dNTP consumption significantly increases as a function of drain$\delta 8$ concentration while a slight decrease is observed for drain$\delta 4$-6. We assume that the trigger tail promotes the dehybridization from drain$\delta 8$ by adopting an ultraloop structure that decreases the Δ_{ext}. As a consequence, the drain template is able to convert more triggers, which explains both the faster dNTP consumption and the reduced amplification rate driven by the replication template. However, this hypothesis has not been thoroughly tested.

In conclusion, we demonstrated the possibility of a drain-based approach with Nt.BstNBI as a nicking enzyme. The optimal parameters $\Delta_{ext}/\Delta_{bind}$ were experimentally determined, and we found that these systems typically required more drain template to reach the bistable regime than those based on Nb.BsmI. We attribute this to the particular arrangement of the recognition and nicking sites for these enzymes, in combination with some leaky activity of Nt.BstNBI on non perfectly hybridized substrates. This is supported by the reactivation mechanism highlighted in Supplementary Fig. 3A.

Supplementary Note 2: Full mathematical models of the bistable autocatalytic loop

The building of a full model for the PEN DNA-toolbox systems has been described in detail in previous publications[4,6]. We first simply add the set of reactions associated with the drain template. Noting *Dr*, *x*, and *xa* respectively the drain template, the autocatalytic active strand and its elongated form, we have:

$$Dr + x \longleftrightarrow x.Dr$$

$$x.Dr \xrightarrow{pol} Dr.xa \qquad \text{(extension by polymerase)}$$

$$Dr + xa \longrightarrow Dr.xa$$

$$xa \xrightarrow{rec} \phi \qquad \text{(degradation by the exonuclease)}$$

We take into account that extended triggers *xa*, after their release from the drain template, can also bind to the autocatalytic template, adding a number of possible adduct species and hybridization reactions to this strand (but no enzymatic reaction, as these triggers cannot be extended nor nicked, at least if we consider the case of the nicking enzyme Nb.BsmI). The complete model for an autocatalytic loop and the associated drain template is shown below:

$$x'(t) = \frac{cpol \cdot kpsd \cdot xTx(t)}{Kmsd \cdot MMf(t)} + cpol \cdot leak \cdot kp \cdot T(t) - k1 \cdot x(t) \cdot (Dr(t) + 2T(t) + Tx(t) + Txa(t) + xaT(t) + xT(t)) + k1 \cdot \left(\frac{xT(t)}{K1i} + \frac{xTx(t) + xTxa(t)}{K1i} + \frac{Tx(t)}{K1o} + \frac{xaTx(t) + xTx(t)}{K1o} \right) - \frac{crec \cdot krec \cdot x(t)}{Kmr \cdot MMr(t)},$$

$$T'(t) = k1 \cdot \left(\frac{xaT(t) + xT(t)}{K1i} + \frac{Tx(t) + Txa(t)}{K1o} \right) - k1 \cdot T(t) \cdot (2x(t) + 2xa(t)),$$

$$xT'(t) = -\frac{cpol \cdot kp \cdot xT(t)}{Km \cdot MMf(t)} - \frac{k1 \cdot xT(t)}{K1i} + \frac{k1 \cdot (xTx(t) + xTxa(t))}{K1o} + k1 \cdot T(t) \cdot x(t) - k1 \cdot xT(t) \cdot (x(t) + xa(t)),$$

$$Tx'(t) = \frac{k1 \cdot (xaTx(t) + xTx(t))}{K1i} - \frac{k1 \cdot Tx(t)}{K1o} + k1 \cdot T(t) \cdot x(t) - k1 \cdot Tx(t) \cdot (x(t) + xa(t)),$$

$$Tx2'(t) = cpol \cdot \left(\frac{kp \cdot xT(t)}{Km \cdot MMf(t)} + \frac{kpsd \cdot xTx(t)}{Kmsd \cdot MMf(t)} \right) - \frac{cn \cdot kn \cdot Tx2(t)}{Kmn \cdot MMn(t)},$$

$$xTx'(t) = \frac{cn \cdot kn \cdot Tx2(t)}{Kmn \cdot MMn(t)} - \frac{cpol \cdot kpsd \cdot xTx(t)}{Kmsd \cdot MMf(t)} - k1 \cdot \left(\frac{1}{K1i} + \frac{1}{K1o} \right) \cdot xTx(t) + k1 \cdot x(t) \cdot (Tx(t) + xT(t)),$$

$$xaT'(t) = -\frac{k1 \cdot xaT(t)}{K1i} + \frac{k1 \cdot (xaTx(t) + xaTxa(t))}{K1o} + k1 \cdot T(t) \cdot xa(t) - k1 \cdot xaT(t) \cdot (x(t) + xa(t)),$$

$$Txa'(t) = \frac{k1 \cdot (xaTxa(t) + xTxa(t))}{K1i} - \frac{k1 \cdot Txa(t)}{K1o} + k1 \cdot T(t) \cdot xa(t) - k1 \cdot Txa(t) \cdot (x(t) + xa(t)),$$

$$xTxa'(t) = -k1 \cdot \left(\frac{1}{K1i} + \frac{1}{K1o} \right) \cdot xTxa(t) + k1 \cdot Txa(t) \cdot x(t) + k1 \cdot xa(t) \cdot xT(t),$$

$$xaTx'(t) = -k1 \cdot \left(\frac{1}{K1i} + \frac{1}{K1o} \right) \cdot xaTx(t) + k1 \cdot Tx(t) \cdot xa(t) + k1 \cdot x(t) \cdot xaT(t),$$

$$xaTxa'(t) = k1 \cdot xa(t) \cdot (Txa(t) + xaT(t)) - k1 \cdot \left(\frac{1}{K1i} + \frac{1}{K1o} \right) \cdot xaTxa(t),$$

$$Dr'(t) = -k1 \cdot Dr(t) \cdot (x(t) + xa(t)) + \frac{k1 \cdot Drxa(t)}{K2} + \frac{k1 \cdot xDr(t)}{K1o},$$

$$xDr'(t) = -\frac{cpol \cdot kp \cdot xDr(t)}{Km \cdot MMf(t)} + k1 \cdot Dr(t) \cdot x(t) - \frac{k1 \cdot xDr(t)}{K1o},$$

$$Drxa'(t) = \frac{cpol \cdot kp \cdot xDr(t)}{Km \cdot MMf(t)} + k1 \cdot Dr(t) \cdot xa(t) - \frac{k1 \cdot Drxa(t)}{K2},$$

$$xa'(t) = -k1 \cdot xa(t) \cdot (Dr(t) + 2T(t) + Tx(t) + Txa(t) + xaT(t) + xT(t)) + k1 \cdot \left(\frac{Drxa(t)}{K2} + \left(\frac{1}{K1i} + \frac{1}{K1o} \right) \cdot xaTxa(t) + \frac{xaT(t) + xaTx(t)}{K1i} + \frac{Txa(t) + xTxa(t)}{K1o} \right) - \frac{crec \cdot krec \cdot xa(t)}{Kmr \cdot MMr(t)},$$

$$MMf(t) = \frac{xT(t)}{Km} + \frac{xTx(t)}{Kmsd} + 1,$$

$$MMn(t) = \frac{Tx2(t)}{Kmn} + 1,$$

$$MMr(t) = \frac{Dr(t)}{Kiexo} + \frac{T(t)}{Kiexo} + \frac{x(t)}{Kmr} + \frac{xa(t)}{Kmr} + 1.$$

The concentrations of enzymes are noted *cpol* (for the polymerase), *crec* (for the exonuclease ttRecJ) and *cn* (for the nickase). The corresponding Michaelis-Menten parameters are given as *kp* and *Km* (*Kmsd* for the reaction with strand displacement) and *krec* and *Kmr*, *kn* and *Kmn*. We take the same exonuclease parameters for the trigger and the extended trigger, as we observed that the degradation kinetics was very similar (probably reflecting the fact that they have the same 5' sequence, given that ttRecJ is a 5'->3' exonuclease). *leak* is a factor representing a constant, leaky production of triggers. *K1o, K1i* and *K2* are the binding constants, respectively of trigger on the output site of the template, trigger on the input site of the template, and extended trigger on the drain. Extended trigger binding constants are taken as identical to trigger constants, which means that we neglect the corresponding dangle effects. These binding constants are calculated for the sequences of the trigger and the drain (e.g. *α* and *drainα*1) using standard thermodynamic values.

We numerically assess the stability of the system by looking at its asymptotic behavior. More precisely we check the existence of a basin of attraction around the 0 state by setting up a small leak, that is, a constant production of triggers. For biochemical realism, this production is set to be proportional to the polymerase concentration and the template concentration (i.e., we assume that the main leak mechanism is un-triggered polymerization of the autocatalytic template; however, this does not affect the generality of the result, as any type of perturbation pushing away from the 0 state would result in amplification for a monostable system).

To build the maps shown in Fig. 2E and 2F (main text), we calculate the binding constants $K1i$ and $K2$ at the working temperature (45 °C) from the binding energies (ΔrG_1 is used to compute $K1o$):

$$\Delta rG_1 i = \Delta rG_2 + \Delta_{\text{bind}}$$
$$\Delta rG_2 = \Delta rG_3 + \Delta_{\text{ext}}$$

We fix the template T total concentration $T(0)$ at 50 nM. Other constants and concentrations are obtained from the literature[4] or individual dedicated measurements (e.g. Michaelis-Menten parameters for the nicking enzyme Nb.BsmI: Vm = 20 nM min^{-1} at 100 U mL^{-1} and Km = 9 nM, see Supplementary Fig. 5). For various combinations of Δ_{bind} and Δ_{ext}, we can then numerically search for the smallest drain concentration that leads to an asymptotically stable 0 state. At this precise concentration, the system (including leak) is marginally stable. We then apply a small additional perturbation sufficient to push it to the basin of attraction of the high state, and record the maximum amplification rate over the trajectory. The range for Δ_{bind} spans approximately the range of destabilization brought by removing 0 to 3 bases from the input side of the template (0.2, 1.0, 1.6 and 2.4 kcal mol^{-1} respectively for 0, 1, 2 and 3 bases in the case of sequence α), while the range of Δ_{ext} values is taken from 2 tailing bases on the drain to 6 and more (for example 1.5, 2.2, 2.9, 3.6 kcal mol^{-1}, respectively for T2, T3, T4, T5 drain tails). Note that those values do not take the destabilizing effect of phosphorothioates into account[3] and that one should assume that the stability of a phosphorothioated drain is systematically smaller than the predicted thermodynamic values for standard backbones.

Fig. 2F shows that the stabilization of the 0 state has a cost in terms of amplification rate. This cost is higher for drains that have a higher turnover rate (i.e. a smaller Δ_{ext}), even if a lower concentration of these drains is required for bistability. Considering the two plots E and F in Fig. 2 (main text), one can define a figure of merit by setting, for example, the acceptable concentration of drain template d_{acc} to be half that of the autocatalytic template (this implies that one does not want to increase excessively the total concentration of DNA in the mixture). With d_{min} the minimal drain concentration required for bistability and $m(d_{\text{min}})$ the maximal amplification observed in these conditions, we then define $M = m(d_{\min})/(d_{acc} + d_{\min})$, leading to the plot in Supplementary Fig. 6A.

Note that the optimal design would in fact depend on the intended use of the bistable switch. The heat map shown in Supplementary Fig. 6B shows the amount of trigger that is needed, as a one-shot injection at time 0, to switch the system from the trivial (0) state to the high state (recall that the minimum drain concentration has been computed to stabilize the 0 state against a *continuous* leaky production of trigger, *not* a step transition in concentration). One sees here that

designs with high Δ_{ext}, which require a high concentration of drain, will need a massive amount of trigger to switch because they will tend to absorb many triggers. Designs with smaller Δ_{ext} should produce a switch that is more sensitive, even if its maximum amplification rate is lower. Other properties that may be optimized include the signal difference between the high and low state (for example if the targeted application involves detection in minute volumes), or the symmetry of the switch operation (if one intends the system to behave as a reversible rewritable memory, back-switching should be approximately symmetrical to the forward switching).

Supplementary Note 3: Toy models with one or two variables

3.1 The behavior of the system changes qualitatively when the drain rate reaches a critical concentration

It is convenient to define a simple empirical one-variable model that combines the three production or degradation processes presented above:

$$\dot{x} = \frac{p \cdot x}{K_p + x} - \deg \cdot x - \frac{drain \cdot x}{K_d + x}$$

Here, p and K_p are the production rate and saturation constant, *deg* the rate of the first order degradation process and *drain* and K_d the rate and saturation constants of the drain degradation pathway. Setting $X = x/K_p$, $\tau = p \cdot t/K_p$, $d = deg \cdot K_p$, $D = K_p \cdot drain/K_d$ and $X_s = K_p/K_d$ we rewrite as:

$$\dot{X} = \frac{X}{1+X} - d \cdot X - D \cdot \frac{X}{1+X/X_s}$$

By searching for the roots of the right hand side, we find the critical value for bistability to be:

$$D_c = 1 - d \qquad \text{with} \qquad X_s < 1 - d$$

For D values above D_c, a new unstable point emerges from the origin, defining the amplification threshold between the two stable points, through a transcritical bifurcation. This shows that to obtain bistability the drain pathway should be fast, but quickly saturable. If D is increased further, the two positive fixed points will vanish through a saddle-node bifurcation, leaving only the origin as attractor: too much degradation forbids amplification. Just above the critical value D_c, we can write $D = D_c + \varepsilon$, and consider that the threshold value for X to leave the null state is small, leading to:

$$\dot{X} = \frac{X}{1+X} - d \cdot X - (1-d+\varepsilon) \cdot \frac{X}{1+X/X_s} \approx \varepsilon \cdot X + \frac{1-d-X_s+\varepsilon}{X_s} \cdot X^2$$

We therefore see that the first order term vanishes when one is sufficiently close to the critical drain rate, leaving an autocatalytic term of second order to drive the initial amplification process:

$$\dot{X} \approx \frac{1-d-X_s}{X_s} X^2$$

On the contrary, well below the critical drain rate, the system is monostable and one can approximate the initial amplification rate at first order as:

$$\dot{X} \approx (1-d-D) \cdot X$$

After integration we obtain that $X(t)/X_{t=0} = e^{(1-d-D)t}$. The observed *Ct* (the time at which the amplification curves cross a given, small value X_e) should then change with the drain rate *D* as $Ct = \ln(X_e/X_0)/(1-d-D) = \frac{A}{1-B \cdot D}$. Hence we expect a hyperbolic signature of the *Ct* as the system evolves to bistability with increasing *D*. Assuming that the drain rate is linearly controlled by the concentration of the drain template we may therefore expect to see the same hyperbolic pattern for the ability of increasing concentrations of drain template to suppress the self-start phenomenon. Then the plot of *1/Ct*, which can be considered as a measure of the self-starting rate, should decrease linearly with the drain template concentration, until it hits the x-axis at the critical drain concentration D_c.

While this analysis is based on a very simple toy model, it is also possible to obtain the same plot from numerical simulations of the complete model presented in section 2. This plot, shown in Supplementary Fig. 7, confirms the hyperbolic signature, at least for large enough Δ_{bind}.

3.2 Delay time (Ct value) as a function of initial trigger concentration in the case of the drain approach

In Supplementary Fig. 8, we numerically perturb the system with various initial concentrations of trigger as a straightforward way to probe its dynamical properties. Indeed, the previous analysis shows that, below the bistable threshold, we should expect a log-linear relationship between *initial* trigger concentration and *Ct* values (as long as the crossing value used to decide *Ct* is sufficiently small so that the first-order approximation used above still holds). On the contrary, above the bistable threshold, we expect an inverse relationship between the two quantities. To show this graphically, we numerically solve the one-variable model described above. Note that a realistic system will be affected by a leak rate, so we need to add the following reaction to the system:

$$\phi \longrightarrow X \quad \text{(leak)}$$

We can see that very different patterns are obtained depending on whether the system is below or above the bistable threshold.

- Below the bistable threshold, we have a classic monostable system, showing the linear $Ct = f(\ln[x]_0)$ relationship for intermediate values of $x_{t=0}$.
- Above the bistable threshold, the spacing of the amplification curves for a logarithmic range of $x_{t=0}$ is not regular anymore, indicative of a non-first-order process. However, we observe a linear relationship between the *Ct* and $1/x_{t=0}$, up to the point where $x_{t=0}$ enters the attraction basin of the null state and the *Ct* value diverges to infinity.

3.3 The dynamical difference between reversible, irreversible (suicide) and irreversible catalytic side-branching pathways

To show the dynamical differences between various side branching scenarios we consider here another slightly more detailed toy model of the system. It contains 3 reactions, accounting respectively for the autocatalytic loop (with Michaelis-Menten kinetics), a first-order decay and the competitive pathway:

$$X \xrightarrow{a} 2X \quad \text{(autocatalysis with Michaelis-Menten kinetics, } K_m = 1\text{)}$$

$$X \xrightarrow{b} \phi \quad \text{(first order decay)}$$

$$X + Y \xrightarrow{c} Z \quad \text{(bimolecular)}$$

A leak is also assumed to produce X at a constant rate k_{leak}. Various options can be envisioned for the outcome of the branching reaction:

- it is reversible, i.e. there is an additional reaction: $Z \xrightarrow{c'} X + Y$
- it is irreversible and non-catalytic (stoichiometric suicide inhibitor): no additional reaction.
- it is irreversible and catalytic (this work). The de-activator *Y* is regenerated: $Z \xrightarrow{c'} Y$

These three cases lead to three very different dynamical situations.

- In the first scenario, the competitive pathway only delays the self-start, and slows down the amplification rate, but never stabilizes the low state. The situation is shown in Supplementary Fig. 9A, where increasing *Y* concentrations linearly relate to an increasing *Ct*. This situation is observed experimentally in Fig. 9 of the main text.
- The second scenario is that of a suicide inhibitor (i.e. a stoichiometric approach). The essential difference with the drain approach is that the drain reactivates itself by releasing its deactivated products – and thus experiences true turnover, while the suicide inhibitor is

consumed by the reaction. As shown in Supplementary Fig. 9B, a suicide inhibitor could significantly delay the self-start process, and put it well beyond experimentally attainable times, but it will not change the overall dynamical properties (i.e. the system remains monostable, but becomes very insensitive as more inhibitor is introduced).

- Finally in the third scenario, of interest here, it is possible to find parameters for which the system becomes bistable above a given $Y_{t=0}$ concentration (Supplementary Fig. 9C). We analyzed this situation from the systems-dynamical point of view in Note 3.1.

Supplementary Fig. 10 illustrates the difference, observed experimentally, between the irreversible, catalytic drain scenario and the irreversible non-catalytic suicide inhibitor scenario. For the latter case, the exonuclease ttRecJ was simply omitted from the solution; the triggers are therefore deactivated by extension on the suicide inhibitor but, as they are not subsequently degraded, they accumulate in the solution and gradually sequester the inhibitor; in this case, the sharp hyperbolic bifurcation to the bistable regime, seen in the drain situation, is lost.

Supplementary Methods

Experimental procedures for experiments with Nt.BstNBI

Reaction mixture assembly: the DNA polymerases Bst 2.0 warm start (8 U μL^{-1}) and Bst full length (5 U μL^{-1}), the nickase Nt.BstNBI (10 U μL^{-1}) and the Bovine Serum Albumin (BSA, 20 mg mL^{-1}) were obtained from New England Biolabs (NEB). A working solution of Bst 2.0 warm start is prepared by dissolving the stock solution 10 times in Diluent A (NEB) complemented with 0.1 % Triton X-100 and stored at -20°C for further use. The thermophilic 5'→3' exonuclease ttRecJ is purified in the laboratory and diluted in Diluent A/0.1 % Triton X-100. All reactions are assembled in a unique buffer containing 45 mM Tris-HCl, 10 mM $(NH_4)_2SO_4$, 10 mM KCl, 50 mM NaCl, 5 mM $MgCl_2$, 2 mM $MgSO_4$, 0.5 mM dithiothreitol (DTT), 0.1 % Synperonic® F 108 (Sigma-Aldrich), 2 μM netropsin. The buffer is prepared as a 4X solution and stored at -20 °C until use. Bulk reactions are assembled at 4 °C in 200 μL PCR test tubes for a final volume of 10 μL and monitored in real-time on a CFX96 or Miniopticon real time PCR thermocycler (Biorad) : the 1X buffer is supplemented with 0.5 X EvaGreen® (Biotium), 3 mM dithiothreitol, dNTPs (50 μM each, NEB), 250 mg mL^{-1} BSA (Molecular Biology Grade, NEB). Enzymes are added just prior to starting the experiment. Experiments from the Supplementary Fig. 2 were performed at 45 °C with 8 U mL^{-1} Bst 2.0 warm start, 200 U mL^{-1} Nt.BstNBI and 25 nM ttRecJ.

The nickase activity toward synthetic extended primers is investigated as follows: the template δtorand (1 μM) is incubated with an equimolar concentration of primer (δ to δ7), together with 0.1 % Nt.BstNBI, 1 % BSA at 42 °C. After a defined incubation time, the nickase is deactivated by heat (80 °C, 30 min). All samples are finally supplemented with 0.5 μL of a mixture of Bst full length (12.5 U mL^{-1} final) / dNTP (200 μM) and incubated for 50 minutes at 42°C, prior to a DNA melting experiment. The derivative of the EvaGreen signal is calculated to determine the melting temperature and the ratio of each species, namely elongated and non-elongated primers.

Supplementary References

References

1. Bommarito, S., Peyret, N., & SantaLucia J. Jr. Thermodynamic parameters for DNA sequences with dangling ends. *Nucleic Acids Res.* **28,** 1929—1934 (2000).
2. SantaLucia, J. Jr. A unified view of polymer, dumbbell, and oligonucleotide DNA nearest-neighbor thermodynamics. *Proc. Natl. Acad. Sci. USA* **95,** 1460—1465 (1998).
3. Stein, C. A., Subasinghe, C., Shinozuka, K. & Cohen, J. S. Physicochemical properties of phosphorothioate oligodeoxynucleotides. *Nucleic Acids Res.* **16,** 3209—3221 (1988).
4. Padirac, A., Fujii, T. & Rondelez, Y. Bottom-up construction of in vitro switchable memories. *Proc. Natl. Acad. Sci. USA* **109,** E3212—E3220 (2012).
5. Hirao, I., Kawai, G., Yoshizawa, S., Nishimura, Y., Ishido, Y., Watanabe, K. & Miura, K. Most compact hairpin-turn structure exerted by a short DNA fragment, d(GCGAAGC) in solution: an extraordinarily stable structure resistant to nucleases and heat. *Nucleic Acids Res.* **22,** 576—582 (1994).
6. Montagne, K., Plasson, R., Sakai, Y., Fujii, T. & Rondelez, Y. Programming an in vitro DNA oscillator using a molecular networking strategy. *Mol. Syst. Biol.* **7,** 466 (2011).
